# Riboflavin-Mediated Photodynamic Therapy in Periodontology: A Systematic Review of Applications and Outcomes

**DOI:** 10.3390/pharmaceutics17020217

**Published:** 2025-02-07

**Authors:** Jakub Fiegler-Rudol, Maciej Łopaciński, Artur Los, Dariusz Skaba, Rafał Wiench

**Affiliations:** Department of Periodontal Diseases and Oral Mucosa Diseases, Faculty of Medical Sciences in Zabrze, Medical University of Silesia, 40-055 Katowice, Poland; mlopacinski@sum.edu.pl (M.Ł.); s79893@365.sum.edu.pl (A.L.); dskaba@sum.edu.pl (D.S.); rwiench@sum.edu.pl (R.W.)

**Keywords:** riboflavin, photodynamic therapy, periodontics, periodontal infections, biofilms, antimicrobial therapy, blue light activation, dental treatments

## Abstract

**Background**: Riboflavin (vitamin B_2_) has emerged as a promising photosensitizer in photodynamic therapy (PDT) due to its strong absorption of blue light and favourable safety profile. This systematic review aims to evaluate the efficacy of riboflavin-mediated PDT in periodontology, specifically examining its antimicrobial effects and potential to improve clinical outcomes compared to conventional or other PDT-based treatments. **Methods**: A systematic review was conducted following PRISMA guidelines. A comprehensive literature search was performed in PubMed/Medline, Embase, Scopus, and the Cochrane Library. Studies published in English within the last 10 years were considered, where riboflavin served as the primary photosensitizer for dental treatments. Data extraction focused on study design, photosensitizer concentration, light source parameters, and clinical or microbiological outcomes. The risk of bias was assessed independently by two reviewers using a predefined scoring system. **Results**: Ten studies met the inclusion criteria, all demonstrating a low risk of bias. Riboflavin-mediated PDT consistently reduced microbial biofilms and pathogen viability in periodontitis, peri-implantitis, and endodontic models. Although some studies reported slightly lower efficacy compared to chlorhexidine or toluidine blue–based PDT, riboflavin-mediated PDT exhibited advantages such as minimal staining, low cytotoxicity, and enhanced collagen crosslinking. However, most studies were in vitro or small-scale clinical trials, limiting conclusions on long-term effectiveness. **Conclusions**: Riboflavin-mediated PDT shows promise as a safe adjunctive therapy for periodontal infections. Larger, well-designed clinical trials with standardized parameters and extended follow-up are needed to further evaluate its efficacy and optimize treatment protocols for routine clinical application.

## 1. Introduction

### 1.1. Rationale

**Figure 1:**
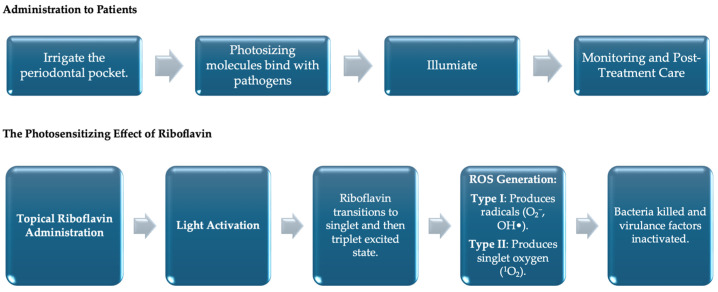
A Schematic representation of clinical and chemical aspects of riboflavin-mediated PDT [4].

Riboflavin, commonly known as vitamin B_2_, is an essential water-soluble vitamin involved in numerous biochemical processes such as cellular metabolism, redox reactions, and the maintenance of tissue integrity [1,2,3]. In addition to its well-known nutritional benefits, riboflavin has gained prominence as a powerful photosensitizing agent in photodynamic therapy (PDT), a minimally invasive treatment modality that is increasingly used in various medical fields, including dentistry [4,5]. Photodynamic therapy integrates three core components: a photosensitizer, light of a specific wavelength, and molecular oxygen [6]. When these elements interact, they generate reactive oxygen species (ROS) that effectively mediate antimicrobial activity, induce cellular damage in pathogens, and modify target tissue structures [4,7]. The duration of action for riboflavin-mediated photodynamic therapy (PDT) depends on the specific treatment protocol, including factors such as the irradiation time, riboflavin concentration, light source used, and the clinical condition being treated [4,7,8,9]. A schematic representation of this is presented in Figure 1.

Riboflavin is ideally suited for PDT because it effectively absorbs blue light and exhibits excellent biocompatibility [8,9]. It shows absorption peaks around 225, 275, 380, and 450 nm. Although these wavelengths do not penetrate deeply into tissues, this limitation may not pose a major issue when bacterial contamination is largely superficial. Additionally, the limited tissue penetration of blue light (405–470 nm) can serve as a benefit by safeguarding patient tissues from extensive photodamage [10]. The gold standard treatment for periodontitis currently involves the combination of systemic and local antibiotic therapies with root planing, effectively targeting pathogenic bacteria and promoting the restoration of periodontal health [7,10,11].

The mechanism of riboflavin-mediated PDT is multifaceted. Upon exposure to light at an appropriate wavelength, riboflavin transitions from its ground state to an excited singlet state, enabling it to interact with molecular oxygen in its environment [11]. This interaction produces ROS, such as singlet oxygen and free radicals, that inflict oxidative damage on bacterial membranes, proteins, and DNA, thereby effectively inactivating pathogens [11,12]. Beyond its antimicrobial effects, photoactivated riboflavin also enhances collagen cross-linking within dentin. This process strengthens the organic matrix and improves the long-term stability of adhesive bonds, making riboflavin-mediated PDT a versatile tool for both microbial control and structural reinforcement in dental procedures [13].

Riboflavin-mediated PDT offers several advantages over traditional dental treatments. Conventional disinfectants such as sodium hypochlorite and chlorhexidine, while effective, carry risks of cytotoxicity and tissue irritation [14,15]. Likewise, techniques like phosphoric acid etching can be invasive and potentially weaken enamel or dentin [16]. In contrast, riboflavin-mediated PDT is minimally invasive, non-toxic, and preserves tissue integrity while delivering robust antimicrobial activity [17]. Moreover, its capacity to stabilize the dentin matrix through collagen cross-linking helps inhibit enzymatic degradation by matrix metalloproteinases (MMPs), which is crucial for maintaining the durability of adhesive bonds [18]. These beneficial properties position riboflavin-mediated PDT as an attractive alternative or adjunct to existing protocols, especially for applications such as disinfecting caries-affected dentin, reconditioning enamel, and enhancing bond strength in adhesive dentistry [15,16,17,18,19].

### 1.2. Objectives

The primary objective of this systematic review is to assess the current evidence regarding the efficacy of riboflavin-mediated PDT in the treatment of various dental conditions. This includes evaluating its antimicrobial effects, its ability to reduce biofilm mass, and its potential to improve clinical outcomes when used alone or in combination with other treatments. Additionally, this review aims to identify the optimal parameters for riboflavin-mediated PDT, including light source specifications and photosensitizer concentrations, to provide evidence-based recommendations for its clinical application. By consolidating findings from in vitro, clinical, and animal studies, this review seeks to highlight the strengths and limitations of riboflavin-mediated PDT, inform future research directions, and potentially facilitate its integration into routine clinical practice.

## 2. Materials and Methods

### 2.1. Focused Question and Null Hypothesis

A systematic review was undertaken using the PICO framework and structured as follows [20]: For patients diagnosed with dental infections or conditions such as peri-implantitis, periodontitis, endodontic infections, or orthodontic biofilms (Population), does the implementation of riboflavin-mediated photodynamic therapy (Intervention), in comparison to conventional treatments such as mechanical debridement, curcumin-mediated PDT, or other non-surgical therapies (Comparison), lead to more effective management, reduced microbial load, or improved clinical outcomes (Outcome)? The null hypothesis for this study posits that there is no significant difference in the effectiveness of managing dental infections or improving clinical outcomes when using riboflavin-mediated photodynamic therapy compared to conventional mechanical debridement, curcumin-mediated PDT, or other non-surgical treatments.

### 2.2. Search Strategy

This systematic review was registered with PROSPERO CRD42025635828 and adhered to the PRISMA 2020 guidelines to ensure clarity and transparency in reporting [21]. A comprehensive search strategy was implemented across several databases, including PubMed/Medline, Embase, Scopus, and the Cochrane Library, using predefined keywords, which are listed in Table 1. Three researchers conducted the database searches independently, employing consistent methodologies. The review included studies published in English between 1 January 2015, and 3 January 2025, based on predefined inclusion criteria. Two authors (J.F-R. and R.W.) screened studies at the title, abstract, and full-text levels. Additionally, reference lists of eligible studies were examined to identify further relevant publications. The findings suggest that photodynamic therapy using riboflavin and curcumin may serve as a promising alternative or complementary approach to conventional peri-implantitis treatments, supported by a careful selection of high-quality studies.

### 2.3. Selection of Studies

In the selection phase of this systematic review, each reviewer independently assessed the titles and abstracts of the retrieved studies to ensure objective inclusion. If disagreements about a study’s eligibility arose, the reviewers collaborated and discussed the matter until a consensus was achieved. This rigorous process, adhering to PRISMA guidelines, ensured that only the most pertinent and methodologically sound studies were included, thereby improving the reliability and reproducibility of the review [21].

The inclusion criteria for this systematic review focused on studies that utilized riboflavin-based PDT for various dental conditions, including both in vitro and animal research. Additionally, research that explored the combined effects of riboflavin-mediated PDT with other antimicrobial or anti-inflammatory treatments was considered. Studies with control groups comparing riboflavin-based PDT to standard mechanical debridement, alternative non-surgical therapies, or no treatment were included, as well as investigations that directly compared the effectiveness of riboflavin-mediated PDT with other non-surgical treatments. Long-term studies assessing the sustained impact of riboflavin-mediated PDT on dental health outcomes, such as infection control, inflammation reduction, and tissue healing, were also included. Only publications that met predefined quality standards (Table 2) and specifically addressed the improvement or management of dental conditions using riboflavin-mediated PDT were considered.

Exclusion criteria included grey and unpublished literature, such as dissertations, conference abstracts, theses, and non-peer-reviewed materials, as well as articles published in languages other than English. Duplicate publications or those with identical ethical approval numbers were excluded. Studies focusing on dental or medical issues outside the scope of this review were also excluded, along with those using alternative photosensitizers other than riboflavin in PDT. Non-relevant in vitro studies that did not replicate clinical dental conditions or address pertinent microbial strains were not included. Furthermore, non-primary data formats such as case reports, case series, narrative reviews, systematic reviews, editorials, and books were excluded, as were studies lacking a control or comparison group. Lastly, studies involving non-therapeutic uses of PDT were not considered.

### 2.4. Risk of Bias in Individual Studies and Quality Assessment

At the start of the study selection process for this systematic review, each reviewer individually examined the titles and abstracts of the identified articles on riboflavin-mediated photodynamic therapy in dental treatments to reduce the risk of bias. The agreement between reviewers was measured using Cohen’s kappa statistic, ensuring consistent and reliable decision-making [22]. In cases where disagreements about a study’s eligibility occurred, the reviewers discussed the issues thoroughly until they reached a unanimous agreement.

The methodological quality of the included studies was independently assessed by two reviewers and focused on critical aspects of riboflavin-mediated photodynamic therapy (PDT) protocols, including study design, execution, and data analysis to maintain objectivity and reliable outcomes. Potential biases were identified by assigning a score of 1 for each “yes” and 0 for each “no” response, based on the following evaluation criteria: (1) Was a specific concentration of riboflavin clearly defined and utilized as the photosensitizer? (2) Was the origin or source of the riboflavin disclosed in the study? (3) Was the incubation period for the riboflavin photosensitizer explicitly stated? (4) Were detailed specifications of the light source provided, including type, wavelength, output power, fluence, and power density? (5) Was a power meter employed to accurately measure the light parameters used in the study? (6) Was a negative control group included in the experimental design to provide a baseline for comparison? (7) Were numerical results presented, accompanied by appropriate statistical analyses? (8) Was there complete reporting of outcome data without any missing information? (9) Funding Independence: Was the study conducted independently of its funding sources to prevent potential conflicts of interest? (10) Were participants and/or investigators blinded to the treatment allocations to minimize bias? Each study’s risk of bias was then classified based on the total number of “yes” responses: High Risk of Bias (0–3 points), Moderate Risk of Bias (4–6 points), and Low Risk of Bias (7–10 points). The total score was calculated for each study, and the corresponding bias risk level (low, moderate, or high) was determined according to the guidelines outlined in the Cochrane Handbook for Systematic Reviews of Interventions [23]. This rigorous quality assessment process ensured that only studies with robust and reliable methodologies were included in the systematic review, thereby enhancing the overall validity and credibility of the review’s findings.

Table 2 provides a detailed evaluation of the risk of bias for the 10 studies included in the final analysis. Inclusion criteria required each study to score at least six points according to the established evaluation framework. All selected studies demonstrated a low risk of bias, with one study achieving the highest possible score of 10 points [24]. Importantly, no studies were classified as having a moderate or high risk of bias, reinforcing the reliability and robustness of the systematic review’s findings.

**Table 2:** The results of the quality assessment and risk of bias across the studies.

Author(s)	1	2	3	4	5	6	7	8	9	10	Total	Risk
Afrasiabi et al., 2023 [25]	1	1	1	1	1	0	1	1	1	0	8	Low
Bärenfaller et al., 2016 [26]	1	1	1	1	1	0	1	1	0	0	7	Low
Comeau et al., 2022 [27]	1	1	1	1	1	0	0	1	1	0	7	Low
Etemadi et al., 2023 [28]	1	1	1	1	1	1	1	1	1	0	9	Low
Kang et al., 2019 [29]	1	1	1	1	1	0	1	1	0	0	7	Low
Kunz et al., 2019 [30]	1	1	1	1	1	1	1	1	0	0	8	Low
Leelanarathiwata et al., 2020 [31]	1	1	1	1	1	1	1	1	0	0	8	Low
Morelato et al., 2022 [32]	1	1	1	1	1	1	1	1	1	0	9	Low
Nielsen et al., 2015 [33]	1	1	1	1	0	1	1	1	1	0	8	Low
Qamar et al., 2023 [24]	1	1	1	1	1	1	1	1	1	1	10	Low

### 2.5. Data Extraction

After finalizing the selection of pertinent studies, the two reviewers extracted detailed information from each included article. This extraction process encompassed bibliographic details such as the lead author’s name and the year of publication, the study design, the specific dental conditions addressed, and the types of experimental and control groups utilized. Additionally, they documented the length of follow-up periods, the outcomes measured related to dental treatment effectiveness, and the technical specifications of the light sources used, including type, wavelength, and power parameters. Furthermore, the reviewers meticulously recorded the concentrations of riboflavin employed as the photosensitizer, the use of nanocarriers or other supplementary agents, as well as the incubation and irradiation durations during photodynamic therapy. They also captured information on any adjunctive treatments or methodologies integrated into the studies. This comprehensive data extraction ensured that all relevant variables were thoroughly captured, enabling a robust and exhaustive analysis of the efficacy and operational parameters of riboflavin-mediated photodynamic therapy in various dental treatments. This systematic approach to data extraction facilitated a nuanced understanding of how riboflavin-mediated PDT performs across different clinical scenarios, thereby strengthening the overall reliability and depth of the systematic review.

## 3. Results

### 3.1. Study Selection

**Figure 2:**
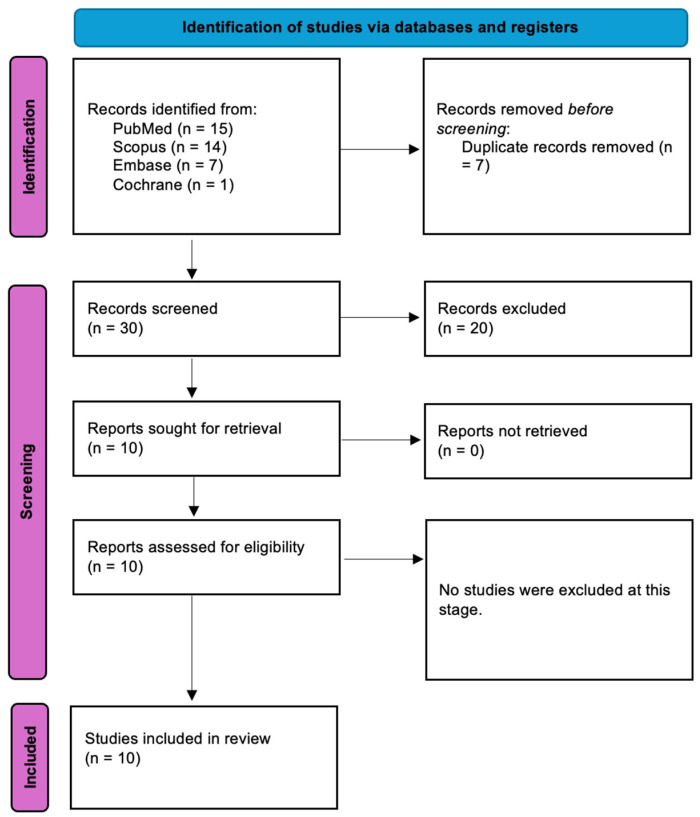
Prisma 2020 flow diagram.

Figure 2 presents an overview of the research workflow, meticulously designed to follow the PRISMA guidelines [23]. The process began with an initial literature search that identified 37 articles. After eliminating duplicates, 30 records remained. A thorough screening of titles and abstracts was conducted, resulting in 10 studies being chosen for a full-text review. Notably, no studies were excluded during this phase. In the end, 10 studies published within the last decade were included in the final analysis. Table 3 provides a detailed summary of these selected studies.

### 3.2. Data Presentation

Data from the 10 studies meeting the inclusion criteria were carefully extracted and are systematically summarized in Table 3, Table 4, Table 5 and Table 6. These tables provide an extensive overview of each study’s key features, including the characteristics of the light sources used and the specific properties of riboflavin employed as a photosensitiser in PDT protocols. This structured presentation enables detailed comparisons and comprehensive analysis of riboflavin-based PDT across the selected studies, supporting a robust assessment of its effectiveness and methodological aspects in addressing dental conditions.

### 3.3. General Characteristics of the Included Studies

The general characteristics of the 10 included studies are shown in Table 3, Table 4, Table 5 and Table 6.

### 3.4. Main Study Outcomes

The reviewed studies underscore the significant potential of riboflavin-mediated PDT for managing microbial biofilms and infections in various periodontal and dental applications. Riboflavin, a biocompatible photosensitizer, generates ROS such as singlet oxygen and hydrogen peroxide when activated by UV or blue light, effectively disrupting microbial cells and biofilms [25,26]. In orthodontic treatments, riboflavin-mediated PDT notably reduced microbial populations around brackets, thereby addressing challenges posed by conventional biofilm management strategies [25]. On implant surfaces, riboflavin-mediated PDT significantly decreased *Aggregatibacter actinomycetemcomitans* biofilms, though its efficacy was slightly lower than chlorhexidine or curcumin-mediated PDT [28,29]. In addition, studies have demonstrated its dose-dependent ability to reduce *Enterococcus faecalis* biofilm formation, with higher riboflavin concentrations and optimized laser parameters improving disinfection outcomes [25]. While results suggest that riboflavin-mediated PDT may be less potent than sodium hypochlorite in some cases, it has the advantage of causing minimal discolouration, indicating its potential as a safer alternative for root canal disinfection [25]. Furthermore, riboflavin-loaded dental resins exhibit enhanced mechanical stability and antimicrobial properties, offering avenues for further optimization [27]. Similar findings on titanium implants indicate that riboflavin-mediated PDT, activated with a 445 nm diode laser, achieves comparable biofilm reductions to methylene blue–based PDT and chlorhexidine, with the added benefit of minimal staining—an important consideration for aesthetic zones [32]. Overall, these results support riboflavin-PDT as a promising adjunctive therapy in periodontology, providing safety, biocompatibility, and material preservation. However, additional research is required to refine its antimicrobial efficacy and determine its value as a standalone clinical treatment.

## 4. Discussion

### 4.1. Results in the Context of Other Evidence

Riboflavin-mediated PDT, activated by blue diode lasers or LEDs, has shown significant antimicrobial effects, particularly at higher light power densities [25]. Although it effectively reduces single-species bacterial counts, its efficacy decreases in complex multi-species biofilms—especially when serum is present—underscoring the importance of adjunctive mechanical biofilm removal [26]. In comparison to the gold standard treatment for periodontitis, which typically involves systemic and local antibiotic administration alongside root planing, riboflavin-mediated PDT offers a targeted antimicrobial strategy that is minimally invasive and may reduce the risk of antibiotic resistance [26,27,28]. Riboflavin-loaded resin systems may facilitate integration into restorative dentistry by providing both antimicrobial activity and mechanical stability [27]. However, their limited penetration into deeper biofilms suggests that additional strategies are required to enhance treatment efficacy in more complex microbial environments. They offer limited penetration into deeper biofilms, which could indicate a need for a photosensitizer that can be excited at a longer wavelength [28]. Riboflavin-mediated PDT also exhibits selective antimicrobial action, effectively reducing certain pathogens such as *Veillonella parvula* but not others, including *Lactobacillus gasseri* [29]. Pre-treatment with hydrogen peroxide significantly boosts its effectiveness, particularly when coupled with mechanical debridement [30]. This combination was particularly effective in reducing complex multi-species biofilms, which are typically more resistant to single-agent treatments. In peri-implantitis, riboflavin-based PDT achieves bacterial reductions comparable to methylene blue–based PDT with red lasers, thus offering a promising option in aesthetically sensitive areas [31,32]. Nonetheless, alternative photosensitizers like toluidine blue O (TBO) under red light may provide more comprehensive microbial eradication in certain clinical scenarios [33]. More recent strategies, such as combining riboflavin-loaded nanoparticles in aloe vera gel with mechanical debridement, further improve clinical outcomes by reducing probing depth and microbial load more effectively than PDT alone [24]. These combinatory approaches highlight the potential for enhanced therapeutic protocols that integrate riboflavin-mediated PDT with other adjunctive treatments to overcome existing limitations. While riboflavin-mediated PDT presents a non-invasive alternative with a lower risk of antibiotic resistance, additional research is necessary to refine treatment protocols, enhance effectiveness in complex biofilms, and assess long-term clinical applications [24,25,26,27,28,29,30,31,32,33,34].

Several recent studies underscore the broad potential of riboflavin-mediated PDT across various dental and medical applications. Łopaciński et al. [14] demonstrated its effectiveness against oral candidiasis, whereas Etemadi et al. [35] found that riboflavin, when photoactivated by blue light, could substantially reduce periodontitis-related bacteria. Notably, Etemadi et al. also reported that blue light alone produced significant bacterial reductions in some cases, highlighting the need for further investigations to delineate riboflavin’s specific contribution as a photosensitizer. In the orthodontic context, Alqerban et al. [36] reported that applying 0.1% riboflavin in PDT effectively bonded orthodontic brackets and conferred considerable antibacterial benefits. Increasing the riboflavin concentration to 0.5% enhanced antimicrobial efficacy yet compromised bonding strength, indicating a trade-off. Kamran et al. [37] similarly confirmed that riboflavin-mediated PDT markedly reduced *Streptococcus mutans* and *Streptococcus sanguinis* levels around orthodontic brackets, underscoring its promise for improving antimicrobial control in orthodontic treatments. Moradi et al. [38] showed that antimicrobial PDT using curcumin or riboflavin significantly reduced *Enterococcus faecalis* biofilms in root canals, achieving comparable results to 5.25% NaOCl. While NaOCl produced the highest bacterial reduction, these findings support PDT as a viable adjunct for root canal disinfection. Beyond oral applications, Najari et al. [39] suggested that riboflavin-mediated PDT, when combined with antibiotics such as colistin, could be valuable for difficult-to-treat *Pseudomonas aeruginosa* infections. Additionally, Alshehri et al. [40] showed that riboflavin-mediated PDT offered the highest antifungal efficacy against *Candida albicans* on acrylic denture surfaces without damaging the denture material. Arboleda et al. [41], however, found that rose bengal–mediated PDT, rather than riboflavin-mediated PDT, effectively inhibited the growth of three fungal species in a corneal infection model. Finally, Kashiwabuchi et al. emphasized that continued refinement in riboflavin preparation and light delivery could further enhance the therapeutic outcomes of riboflavin-mediated PDT, reinforcing the need for additional in vitro and clinical trial [42]. While in situ gels like Atridox provide sustained doxycycline release without the need for additional equipment, riboflavin-mediated PDT requires photoactivation but offers selective antimicrobial action and potential integration into restorative dental materials, and further research is needed to fully evaluate and optimize both approaches for managing complex periodontal infections [43,44,45,46,47]. The integration of riboflavin-mediated PDT into various clinical practices demonstrates significant potential, yet highlights the necessity for ongoing optimization and validation through further research [23,24,25,26,27,28,29,30,31,32,33,34,35,36,37,38,39,40,41,42,43,44].

### 4.2. Limitations of the Evidence

Despite the promising outcomes reported, several limitations in the current body of evidence constrain a definitive assessment of riboflavin-mediated PDT in periodontology. First, the marked heterogeneity in study protocols, ranging from the type and intensity of light sources to the concentration of riboflavin and the duration of exposure, makes direct comparisons challenging and precludes the establishment of standardized guidelines [21,24,25,26,27,28,29,30,31,32,33]. Additionally, most of the included studies were in vitro, limiting the generalizability of their findings to clinical practice [24,25,26,27,28,29,30,31,32,33]. Even among the few in vivo or clinical trials, sample sizes were frequently small and follow-up times relatively short, raising questions about long-term efficacy and safety [24,25,26,27,28,29,30,31,32,33]. Moreover, outcome measures often relied on colony-forming units or visually assessed biofilm reduction without consistent use of advanced or standardized diagnostic tools [24,25,26,27,28,29,30,31,32,33]. Two major issues are the scattering of short-wavelength light, which limits efficacy, and the possibility that photokilling a portion of the cell population could lead to the development of an unresponsive group of cells, which were not addressed by the studies analysed [29,33]. Finally, potential biases, such as the lack of blinding and reliance on subjective endpoints, further highlight the need for more robust, controlled clinical trials with uniform protocols, larger cohorts, and longer observation periods [21,22,23].

### 4.3. Limitations of the Review Process

A key limitation of the review process relates to the marked heterogeneity of study designs, intervention protocols, and outcome measures, which necessitated a largely narrative synthesis rather than a quantitative meta-analysis. While PRISMA guidelines were followed to enhance transparency, the exclusion of non-English studies and grey literature potentially narrowed the scope, increasing the risk of missing relevant data. Furthermore, although study selection and data extraction were conducted in duplicate, the reliance on authors’ subjective judgments may introduce inadvertent bias. Finally, the decision not to apply the GRADE framework, due to substantial variability in parameters and methodologies, limits the ability to draw definitive conclusions about the overall certainty of the evidence. These factors highlight the need for more uniform protocols and comprehensive strategies in future systematic reviews of riboflavin-mediated photodynamic therapy in periodontology.

### 4.4. Implications for Practice, Policy, and Future Research

Riboflavin-mediated PDT shows considerable promise as an adjunctive or alternative strategy in periodontal care, especially for managing biofilm-related infections and potentially reducing reliance on conventional antimicrobials. Clinically, practitioners could integrate riboflavin-mediated PDT into existing protocols for periodontal debridement or implant maintenance, leveraging its generally low toxicity and minimal staining profile compared to other photosensitizers. From a policy perspective, regulatory bodies and professional organizations should consider supporting standardized protocols and guidelines to facilitate consistent implementation across different clinical settings. This includes establishing optimal light parameters, riboflavin concentrations, and treatment durations to maximize therapeutic efficacy. In parallel, future research must focus on high-quality randomized controlled trials with robust sample sizes and longer follow-up to validate riboflavin-mediated PDT’s effectiveness in vivo. Investigations into combination therapies, such as pairing PDT with other antimicrobial or anti-inflammatory agents and innovations in photosensitizer delivery methods (e.g., nanoparticles or bioactive materials) may further enhance treatment outcomes. Through these combined efforts, riboflavin-mediated PDT stands to gain broader acceptance and become an integral component of evidence-based periodontal care.

## 5. Conclusions

The findings of this systematic review suggest that riboflavin-mediated PDT holds considerable promise for enhancing antimicrobial efficacy and clinical outcomes in periodontology. Although the heterogeneity in study designs and outcome measures precludes a definitive consensus, the cumulative evidence indicates that riboflavin-mediated PDT often compares favourably, or at least equivalently, to conventional interventions such as mechanical debridement. Consequently, while the results do not uniformly or unequivocally refute the null hypothesis, which posits no significant difference in effectiveness between riboflavin-mediated PDT and established non-surgical therapies, they do increasingly challenge it. Notably, riboflavin-mediated PDT demonstrated advantages in reducing biofilm mass, minimizing staining, and potentially lowering the risk of antibiotic resistance. To provide more conclusive guidance for clinical practice, future well-designed randomized controlled trials with standardized protocols, larger cohorts, and extended follow-up periods are needed to fully determine whether riboflavin-mediated PDT indeed offers a superior or distinct therapeutic benefit in periodontal care.

## Figures and Tables

**Table 1:** Search syntax used in the study.

Source	Search Term	Filters	Number of Results
Medline via PubMed	(riboflavin OR “vitamin B2”) AND (“photodynamic therapy” OR photochemotherapy OR phototherapy OR PDT OR “light-activated disinfection” OR photosensitizer OR photosensitizing OR “light therapy”) AND (periodontal OR periodontics OR periodontology OR periodontitis OR periodont* OR gingivitis OR “gum disease” OR “gum infection”)	2015–2025	15
Web of Science Scopus	(TITLE-ABS-KEY (riboflavin OR “vitamin B2”)) AND (TITLE-ABS-KEY(“photodynamic therapy” OR photochemotherapy OR phototherapy OR PDT OR “light-activated disinfection” OR photosensitizer OR photosensitizing OR “light therapy”)) AND (TITLE-ABS-KEY (periodontal OR periodontics OR periodontology OR periodontitis OR periodont* OR gingivitis OR “gum disease” OR “gum infection”))	2015–2025	14
Embase	((‘riboflavin’: ti, ab, kw OR ‘vitamin B2’: ti, ab, kw)) AND ((‘photodynamic therapy’: ti, ab, kw OR photochemotherapy: ti, ab, kw OR phototherapy: ti, ab, kw OR pdt: ti, ab, kw OR ‘light-activated disinfection’: ti, ab, kw OR photosensitizer: ti, ab, kw OR photosensitizing: ti, ab, kw OR ‘light therapy’: ti, ab, kw)) AND ((periodontal: ti, ab, kw OR periodontics: ti, ab, kw OR periodontology: ti, ab, kw OR periodontitis: ti, ab, kw OR periodont*: ti, ab, kw OR gingivitis: ti, ab, kw OR ‘gum disease’: ti, ab, kw OR ‘gum infection’: ti, ab, kw))	2015–2025	7
Cochrane database	(MH “Riboflavin” OR “Riboflavin” OR “Vitamin B2”) AND (MH “Photodynamic Therapy” OR “Photodynamic Therapy” OR “PDT” OR “Photochemotherapy” OR “Antimicrobial Photodynamic Therapy” OR “Light-Activated Disinfection” OR “Photosensitizer” OR “Photosensitizing” OR “Light Therapy”) AND (MH “Dentistry” OR MH “Oral Health” OR “Periodontal” OR “Periodontics” OR “Periodontology” OR “Periodontitis” OR “Periodont*” OR “Gingivitis” OR “Gum Disease” OR “Gum Infection”)	2015–2025	1

**Table 3:** Summary of the studies.

Author and Year	Country	Study Design	Programs Used (Version, Specifications, Model, Brand, Company, City, State, Country)
Afrasiabi et al. 2023 [25]	Iran	in vitro study	Blue diode laser (0.4–1.0 W/cm^2^), Curcumin and Riboflavin, Chlorhexidine, Laser Research Center of Dentistry, Tehran University of Medical Sciences, Tehran, Iran
Bärenfaller et al., 2016 [26]	Switzerland	in vitro study	Photoactivated disinfection using LED (Blue and Red), Riboflavin, University of Bern, Freiburgstrasse, Bern, Switzerland
Comeau et al., 2022 [27]	Canada	in vitro study	Blue LED (1.3 J/cm^2^), Riboflavin-loaded dental resin, University of British Columbia, Vancouver, Canada
Etemadi et al., 2023 [28]	Iran	in vitro study	LED irradiation (390–480 nm), Curcumin and Riboflavin, Chlorhexidine 0.12%, Tehran Medical Sciences, Islamic Azad University, Tehran, Iran
Kang et al., 2019 [29]	South Korea	in vitro study	405 nm blue light (25.3 J), Curcumin, Protoporphyrin-IX, Resazurin, Riboflavin, Yonsei University College of Dentistry, Seoul, South Korea
Kunz et al., 2019 [30]	Switzerland	in vitro study	Blue LED (30 s, 60 s), Riboflavin, Hydrogen Peroxide, University of Bern, Switzerland
Leelanarathiwata et al., 2020 [31]	Thailand	in vitro study	Blue high-power LED, Flavin Mononucleotide, Mahidol University, Bangkok, Thailand
Morelato et al., 2022 [32]	Croatia	in vitro study	445 nm diode laser, Riboflavin, University of Rijeka, Croatia
Nielsen et al., 2015 [33]	Denmark	in vitro study	Blue LED, Riboflavin and Toluidine Blue O, Aarhus University, Denmark
Qamar et al., 2023 [24]	Saudi Arabia	Randomized controlled trial	Riboflavin-loaded Poly-L-glycolic acid nanoparticles, Aloe Vera gel, Riyadh Elm University, Riyadh, Saudi Arabia (Marked in Blue)

**Table 4:** Main outcomes and study groups.

Author and Year	Study Groups	Main Outcomes
Afrasiabi et al., 2023 [25]	Negative Control: No treatment. Positive Control: Bacterial suspension exposed to CHX at 25 °C for 1 min. Curcumin Only: Bacterial suspension exposed to curcumin (40 µM) in the dark at 25 °C for 5 min. Riboflavin Only: Bacterial suspension exposed to riboflavin (100 µM) in the dark at 25 °C for 5 min. aPDT with Curcumin + Blue Diode Laser aPDT with Riboflavin + Blue Diode Laser	Riboflavin-mediated PDT demonstrates significant bactericidal effects against periodontal pathogens like *Streptococcus mutans*, particularly when combined with a blue diode laser at optimized power densities. The study found that riboflavin, activated by 445 nm blue light, induces oxidative stress, leading to microbial decontamination. The bactericidal efficacy improves with increased laser power density, with maximum effect observed at 1.0 W/cm^2^. Compared to conventional antimicrobial agents such as chlorhexidine, riboflavin-mediated PDT offers a non-invasive alternative, reducing bacterial colonies without systemic side effects, thus enhancing its potential as a sustainable periodontal therapy.
Bärenfaller et al., 2016 [26]	Negative Control: No treatment. Light Control: light only. Photosensitizer Control: Without light. PAD30 (Blue LED): Photoactivated disinfection with blue LED for 30 s with riboflavin as photosensitizer. PAD60 (Blue LED): Photoactivated disinfection with blue LED for 60 s with riboflavin as photosensitizer. PADred: Photoactivated disinfection with red LED for 60 s with toluidine blue as photosensitizer.	Riboflavin-mediated PDT, often combined with blue spectrum LED, demonstrates notable antimicrobial effects against periodontopathogenic species. Studies highlight its efficacy in reducing planktonic bacterial counts, including pathogens such as *Porphyromonas gingivalis* and *Aggregatibacter actinomycetemcomitans*. However, the bactericidal impact is time and dose-dependent, with increased exposure enhancing outcomes. Riboflavin-mediated PDT shows diminished efficacy in complex multi-species biofilms and in the presence of serum, underlining the importance of mechanical biofilm removal as an adjunct. Despite its promising potential, riboflavin-mediated PDT’s effectiveness is generally inferior to red-spectrum light systems using toluidine blue. Its clinical relevance lies in supplementing traditional periodontal therapies while minimizing antibiotic resistance risks
Comeau et al., 2022 [27]	Control Groups: Resin blend without riboflavin (0% B_2_). Bacterial biofilm-only group (*Streptococcus mutans*) without resin disks. Experimental Resin Groups: Riboflavin-loaded resins with concentrations: 0.1%, 1.0%, and 1.5% B_2_. Light Treatment Sub-Groups: Dark: No light exposure. Light: Exposure to blue LED light (440–460 nm) for 60 s at a fluence of 1.3 J/cm^2^. Aging Conditions: Immediate: Resin specimens sterilized and tested immediately after fabrication. Aged: Resin specimens stored in 37 °C ultrapure water for 28 days before testing.	Riboflavin-mediated photodynamic therapy (PDT) in periodontology demonstrates significant antimicrobial and restorative potential. As a photosensitizer, riboflavin is activated by blue light to produce ROS that effectively reduce bacterial viability, particularly against pathogens like *Porphyromonas gingivalis*, *Aggregatibacter actinomycetemcomitans*, and *Streptococcus mutans*. Its efficacy is enhanced with optimized light parameters, such as higher fluence rates and appropriate exposure times, though it is less effective against complex biofilms and in the presence of serum. Studies show that riboflavin-mediated PDT can decrease biofilm formation on dental materials and integrate into resin-based restorations, providing mechanical stability and antimicrobial activity without compromising material properties. Further optimization of concentration, light parameters, and biofilm models is necessary to maximize its clinical applicability.
Etemadi et al., 2023 [28]	Negative Control: No treatment Positive Control: 0.12% chlorhexidine (5 s) Curcumin Group: 5 mg/mL curcumin in the dark for 5 min. Riboflavin Group: 0.5% riboflavin in the dark for 5 min. LED Group: Exposed to blue light (390–480 nm) for 60 s. Curcumin-mediated PDT: 5 mg/mL + LED. Riboflavin-mediated PDT: 0.5% + LED.	Riboflavin-mediated PDT in periodontology and peri-implantology exhibits promising antimicrobial effects, especially against *Aggregatibacter actinomycetemcomitans*. When activated by blue light or LEDs in the 390–480 nm range, riboflavin generates ROS that disrupt bacterial membranes, reducing biofilm viability on teeth and implant surfaces. Riboflavin mediated PDT effectively lowers bacterial CFU/mL in vitro, with better results under optimized conditions such as higher light intensity and adequate exposure time. Although generally less effective than curcumin-mediated PDT or chlorhexidine, riboflavin-mediated PDT offers a biocompatible, non-invasive alternative that avoids systemic side effects and minimizes antibiotic resistance risks. While its ability to kill biofilms is clear, clinical use may be limited by complex biofilm structures and low oxygen levels in deep periodontal pockets.
Kang et al., 2019 [29]	4 photosensitizers tested: Curcumin: 100 μg/mL, 10 μg/mL, 1 μg/mL, and 0.1 μg/mL. Protoporphyrin IX: Same concentrations as curcumin. Resazurin: Same concentrations as curcumin. Riboflavin: Same concentrations as curcumin. Light Exposure: 405 nm violet-blue LED irradiation (25.3 J). Dark Condition: No light exposure (PS only).	Riboflavin-mediated PDT demonstrated antimicrobial effects against *Veillonella parvula* but showed limited or no activity against *Lactobacillus gasseri* at lower concentrations. Its efficacy varied across bacterial strains, emphasizing its potential for selective bacterial inhibition in periodontal therapy while highlighting the need for optimization in biofilm models and clinical applications.
Kunz et al., 2019 [30]	Control Group: 0.9% NaCl. Riboflavin (0.1%) + 30 s of blue LED light. Riboflavin (0.1%) + 60 s of blue LED light. 0.25% H_2_O_2_ + Riboflavin + 30 s of blue LED light. 0.25% H_2_O_2_ + Riboflavin + 60 s of blue LED light. 3% H_2_O_2_ + Riboflavin + 30 s of blue LED light. 3% H_2_O_2_ + Riboflavin + 60 s of blue LED light. Mechanical biofilm removal only. Mechanical biofilm removal + 0.25% H_2_O_2_ + Riboflavin + 60 s of blue LED light. Mechanical biofilm removal + 3% H_2_O_2_ + Riboflavin + 60 s of blue LED light.	It demonstrates that while aPDT alone has limited activity against biofilms, its efficacy significantly increases when preceded by H_2_O_2_ pretreatment, particularly at a 3% concentration. This combined approach effectively eliminates planktonic bacteria and multi-species biofilms, with no detectable bacteria remaining when coupled with mechanical biofilm removal. This method could provide a potent alternative to traditional antimicrobial periodontal therapies, reducing the reliance on antibiotics and their associated resistance issues.
Leelanarathiwata et al., 2020 [31]	Control Group: No treatment. Light Irradiation Only Groups: Red diode laser (for 60 s). Blue high-power LED (for 10 s). Red Laser + Methylene Blue Blue LED + FMN	Riboflavin-mediated PDT, employing flavin mononucleotide activated by blue high-power LED light, demonstrates significant antibacterial efficacy against *Staphylococcus aureus* biofilms, a key contributor to peri-implantitis. This novel approach yields bacterial reductions comparable to established methylene blue–based PDT with red laser activation, achieving over 93% bacterial reduction. Riboflavin’s natural presence and safer profile, combined with shorter treatment durations and effective biofilm penetration, position it as a promising alternative or adjunctive treatment to conventional scaling, root planing, and antibiotics, particularly amidst rising antibiotic resistance. While both PDT systems effectively reduce biofilm mass and bacterial viability, riboflavin’s activation by blue light offers aesthetic and operational advantages, though further optimization and studies are needed for deeper tissue applications and enhanced biofilm eradication.
Morelato et al., 2022 [32]	Negative Control: Untreated implants. Positive Control: Implants treated with 0.2% CHX s for 60 s using brushing movements. PDT Groups: PDT1 (660 nm diode laser): Methylene blue (0.1%) as a photosensitizer, activated with a 660 nm diode laser for 60 s. PDT2 (445 nm diode laser): Riboflavin (0.1%) as a photosensitizer, activated with a 445 nm diode laser for 60 s.	Riboflavin-mediated PDT, specifically utilizing a 445 nm diode laser combined with 0.1% riboflavin as a photosensitizer, demonstrates significant efficacy in reducing biofilms of *Staphylococcus aureus* and *Candida albicans* on dental implant surfaces. This method shows comparable outcomes to conventional 660 nm diode laser PDT with methylene blue and 0.2% CHX treatments, without significant differences in microbial reduction among these methods. Advantages include minimal aesthetic disruption due to riboflavin’s light-yellow colour, which contrasts with the staining caused by methylene blue. The technique proves to be a promising adjunct to mechanical debridement for peri-implantitis, particularly in aesthetic zones, although it does not completely eradicate all microorganisms or organic material.
Nielsen et al., 2015 [33]	Negative Control Group (P−L−): Microbial suspension treated with sterile saline without light or photosensitizer. Light Alone Group (P−L+): Microbial suspension irradiated with light (red or blue LED) without photosensitizer. Photosensitizer Alone Group (P+L−): Microbial suspension treated with photosensitizer without light. Photoactivated Disinfection (PAD) Groups (P+L+): Riboflavin + Blue Light: Treated with riboflavin and irradiated with 460 nm blue LED. Toluidine Blue O + Red Light: Treated with toluidine blue O and irradiated with 630 nm red LED.	Riboflavin-mediated PDT with blue light showed limited antimicrobial effects compared to TBO with red light for periodontal and endodontic pathogens. Although riboflavin effectively reduced *Porphyromonas gingivalis* and *Prevotella intermedia*, similar reductions occurred with blue light alone, indicating activation of bacterial endogenous chromophores. For other pathogens, riboflavin only caused minor to moderate bacterial reductions, and longer irradiation times did not enhance efficacy within clinical limits. In contrast, TBO-mediated PAD completely eradicated all tested pathogens even with short irradiation, demonstrating superior antimicrobial efficacy. Thus, riboflavin is unsuitable as a photosensitizer for periodontal or endodontic infections, while TBO shows significant promise.
Qamar et al., 2023 [24]	Group 1: PGA/RF/AV + MD Group 2: PDT + MD Group 3: MD Alone	While PDT alone significantly reduced BoP and microbial loads of Tf, the application of PGA/RF/AV demonstrated better outcomes. This compound significantly improved PD, PI, and MBL, with enhanced reductions in microbial counts of both *Porphyromonas gingivalis* and Tf. These findings highlight the potential of the PGA/RF/AV complex as a more effective treatment modality than PDT alone, leveraging the bactericidal and anti-inflammatory properties of riboflavin and aloe vera to achieve improved clinical and microbiological outcomes.

aPDT—Antimicrobial Photodynamic Therapy; AV—Aloe Vera; B_2_—Vitamin B_2_ (Riboflavin); CFU—Colony-Forming Unit(s); CHX—Chlorhexidine; FMN—Flavin Mononucleotide; H_2_O_2_—Hydrogen Peroxide; J—Joule; LED—Light-Emitting Diode; MD—Mechanical Debridement; MBL—Marginal Bone Level; PD—Probing Depth; PGA—Poly-L-Glycolic Acid; PI—Plaque Index; PDT—Photodynamic Therapy; P−L−—No Photosensitizer and No Light; P+L−—Photosensitizer Only; P−L+—Light Only; P+L+—Photoactivated Disinfection; PS—Photosensitizer; RF—Riboflavin; TBO—Toluidine Blue O; Tf—*Tannerella forsythia*; BoP—Bleeding on Probing; PGA/RF/AV—Riboflavin-Loaded Poly-L-Glycolic Acid Nanoparticles in Aloe Vera Gel; PI—Plaque Index; MBL—Marginal Bone Level.

**Table 5:** Summary of light source parameters from each study.

Light Source	Author/Year	Operating Mode	Wavelength (nm)	Energy Density (Fluence) (J/cm^2^)	Power Output (mW)	Irradiation Time (s)
Blue diode laser (Pioon, China)	Afrasiabi et al., 2023 [25]	Continuous	445 nm	12, 18, 24, and 30	-	30 s
FotoSan460; CMS Dental ApS, Copenhagen, Denmark	Bärenfaller et al., 2016 [26]	Continuous	460 ± 10	120	1	30, 60
BioLight (Department of Physics and Astronomy at the University of British Columbia, Vancouver, BC, Canada)	Comeau et al., 2022 [27]	Continuous	440–460	1.3	830	60
LED (DY400-4, Denjoy, China)	Etemadi et al., 2023 [28]	Continuous	390–480	300–420	1000 ± 100	-
QLF-D (s digital, Inspektor Research Systems, Amsterdam, The Netherlands)	Kang et al., 2019 [29]	Continuous	405	25.3	84.5	-
FotoSan460; CMS Dental ApS, Copenhagen, Denmark).	Kunz et al., 2019 [30]	Continuous	460 ± 10	-	1	30, 60
FotoSan^®^ BLUE LAD; (CMS Dental, Denmark)	Leelanarathiwata et al., 2020 [31]	Continuous	450–470	37–40	29.1−31.4	10
Diode laser (SiroLaser Blue, Dentsply Sirona, Bensheim, Germany)	Morelato et al., 2022 [32]	Pulsed mode (100 Hz)	445	1.24	100	60
FotoSan 630 LAD pen FlashMax P3 460 (CMS Dental, Denmark)	Nielsen et al., 2015 [33]	Continuous	630 460	37.7	0.4	60
Diode laser system (Periowave™ Vancouver, BC, Canada)	Qamar et al., 2023 [24]	Continuous	670	-	280	60

LED—Light-Emitting Diode; QLF-D—Quantitative Light-Induced Fluorescence Device.

**Table 6:** Summary of photosynthesizer concentrations and incubation times.

Author and Year	Concentration/s of Riboflavin Used
Afrasiabi et al., 2023 [25]	100 µM
Bärenfaller et al., 2016 [26]	0.1%
Comeau et al., 2022 [27]	0.1–1.5%
Etemadi et al., 2023 [28]	0.5%
Kang et al., 2019 [29]	10 µg/mL
Kunz et al., 2019 [30]	0.1%
Leelanarathiwata et al., 2020 [31]	0.18 FMN
Morelato et al., 2022 [32]	0.1%
Nielsen et al., 2015 [33]	133 μmol/L inplanktonic microbial suspension
Qamar et al., 2023 [24]	0.1%

FMN-riboflavin-5′-phosphate.

## References

[1] Peechakara B.V., Sina R.E., Gupta M. (2025). Vitamin B2 (Riboflavin) [Updated 1 February 2024]. StatPearls [Internet].

[2] Averianova L.A., Balabanova L.A., Son O.M., Podvolotskaya A.B., Tekutyeva L.A. (2020). Production of Vitamin B2 (Riboflavin) by Microorganisms: An Overview. Front. Bioeng. Biotechnol..

[3] Powers H.J. (2003). Riboflavin (vitamin B2) health. Am. J. Clin. Nutr..

[4] Insińska-Rak M., Sikorski M., Wolnicka-Glubisz A. (2023). Riboflavin and Its Derivates as Potential Photosensitizers in the Photodynamic Treatment of Skin Cancers. Cells.

[5] Niculescu A.G., Grumezescu A.M. (2021). Photodynamic Therapy—An Up-to-Date Review. Appl. Sci..

[6] Correia J.H., Rodrigues J.A., Pimenta S., Dong T., Yang Z. (2021). Photodynamic Therapy Review: Principles, Photosensitizers, Applications, and Future Directions. Pharmaceutics.

[7] Alfei S., Schito G.C., Schito A.M., Zuccari G. (2024). Reactive Oxygen Species (ROS)-Mediated Antibacterial Oxidative Therapies: Available Methods to Generate ROS and a Novel Option Proposal. Int. J. Mol. Sci..

[8] Azizi A., Shohrati P., Goudarzi M., Lawaf S., Rahimi A. (2019). Comparison of the effect of photodynamic therapy with curcumin and methylene Blue on streptococcus mutans bacterial colonies. Photodiagnosis Photodyn. Ther..

[9] Pordel E., Ghasemi T., Afrasiabi S., Benedicenti S., Signore A., Chiniforush N. (2023). The Effect of Different Output Powers of Blue Diode Laser along with Curcumin and Riboflavin against *Streptococcus mutans* around Orthodontic Brackets: An In Vitro Study. Biomedicines.

[10] Schlafer S., Vaeth M., Hørsted-Bindslev P., Frandsen E.V. (2010). Endodontic photoactivated disinfection using a conventional light source: An in vitro and ex vivo study. Oral Surg. Oral Med. Oral Pathol. Oral Radiol. Endodontology.

[11] Abrahamse H., Hamblin M.R. (2016). New photosensitizers for photodynamic therapy. Biochem. J..

[12] Kashef N., Hamblin M.R. (2017). Can microbial cells develop resistance to oxidative stress in antimicrobial photodynamic inactivation?. Drug Resist. Updat..

[13] Chen H., Sun G., Wang H., Yu S., Tian Z., Zhu S. (2023). Effect of collagen cross-linkers on dentin bond strength: A systematic review and network meta-analysis. Front. Bioeng. Biotechnol..

[14] Łopaciński M., Fiegler-Rudol J., Niemczyk W., Skaba D., Wiench R. (2025). Riboflavin- and Hypericin-Mediated Antimicrobial Photodynamic Therapy as Alternative Treatments for Oral Candidiasis: A Systematic Review. Pharmaceutics.

[15] Khoury R.D., de Carvalho L.S., do Nascimento M.F.R., Alhussain F., Abu Hasna A. (2024). Endodontic irrigants from a comprehensive perspective. World J. Clin. Cases.

[16] Pashley D.H., Tay F.R., Breschi L., Tjäderhane L., Carvalho R.M., Carrilho M., Tezvergil-Mutluay A. (2011). State of the art etch-and-rinse adhesives. Dent. Mater..

[17] De Silva P., Saad M.A., Thomsen H.C., Bano S., Ashraf S., Hasan T. (2020). Photodynamic therapy, priming and optical imaging: Potential co-conspirators in treatment design and optimization—A Thomas Dougherty Award for Excellence in PDT paper. J. Porphyr. Phthalocyanines.

[18] de Moraes I.Q.S., do Nascimento T.G., da Silva A.T., de Lira L.M.S.S., Parolia A., Porto I.C.C.M. (2020). Inhibition of matrix metalloproteinases: A troubleshooting for dentin adhesion. Restor. Dent. Endod..

[19] Alrefeai M., Aljamhan A., Al Habdan A.H., Alzehiri M., Naseem M., Alkhudhairy F. (2022). Influence of methylene blue, riboflavin, and indocyanine green on the bond strength of caries-affected dentin when bonded to resin-modified glass ionomer cement. Photodiagnosis Photodyn. Ther..

[20] Schardt C., Adams M.B., Owens T., Keitz S., Fontelo P. (2007). Utilization of the PICO Framework to Improve Searching PubMed for Clinical Questions. BMC Med. Inform. Decis. Mak..

[21] Page M.J., McKenzie J.E., Bossuyt P.M., Boutron I., Hoffmann T.C., Mulrow C.D., Shamseer L., Tetzlaff J.M., Akl E.A., Brennan S.E. (2021). The PRISMA 2020 statement: An updated guideline for reporting systematic reviews. BMJ.

[22] Watson P.F., Petrie A. (2010). Method Agreement Analysis: A Review of Correct Methodology. Theriogenology.

[23] Higgins J.P.T., Thomas J., Chandler J., Cumpston M., Li T., Page M.J., Welch V.A. (2019). Cochrane Handbook for Systematic Reviews of Interventions.

[24] Qamar Z., Sayed Abdul N., Soman C., Shenoy M., Bamousa B., Rabea S., Albahkaly H.S. (2023). Clinical and radiographic peri-implant outcomes with riboflavin-loaded Poly-L-glycolic acid nanoparticles incorporated in aloe-vera gel treating peri-implantitis in chronic hyperglycemic patients. Photodiagnosis Photodyn. Ther..

[25] Afrasiabi S., Entezari S., Etemadi A., Chiniforush N. (2023). The influence of different modes of power density during antimicrobial photodynamic therapy for photokilling of *Streptococcus mutans*. Photodiagnosis Photodyn. Ther..

[26] Bärenfaller V., Clausen C., Sculean A., Eick S. (2016). Effect of photoactivated disinfection using light in the blue spectrum. J. Photochem. Photobiol. B.

[27] Comeau P., Burgess J., Rezqi Qomi N., Lee A., Manso A. (2022). The antimicrobial, physical, and chemical properties of a riboflavin-loaded dental resin intended for antimicrobial photodynamic therapy. Photodiagnosis Photodyn. Ther..

[28] Etemadi A., Hashemi S.S., Chiniforush N. (2023). Evaluation of the effect of photodynamic therapy with curcumin and riboflavin on implant surface contaminated with *Aggregatibacter actinomycetemcomitans*. Photodiagnosis Photodyn. Ther..

[29] Kang S.M., Jung H.I., Kim B.I. (2019). Susceptibility of oral bacteria to antibacterial photodynamic therapy. J. Oral Microbiol..

[30] Kunz D., Wirth J., Sculean A., Eick S. (2019). In-vitro activity of additive application of hydrogen peroxide in antimicrobial photodynamic therapy using LED in the blue spectrum against bacteria and biofilm associated with periodontal disease. Photodiagnosis Photodyn. Ther..

[31] Leelanarathiwata K., Katsuta Y., Katsuragi H., Watanabe F. (2020). Antibacterial activity of blue high-power light-emitting diode-activated flavin mononucleotide against *Staphylococcus aureus* biofilm on a sandblasted and etched surface. Photodiagnosis Photodyn. Ther..

[32] Morelato L., Budimir A., Smojver I., Katalinić I., Vuletić M., Ajanović M., Gabrić D. (2022). A novel technique for disinfection treatment of contaminated dental implant surface using 0.1% riboflavin and 445 nm diode laser—An in vitro study. Bioengineering.

[33] Nielsen H.K., Garcia J., Væth M., Schlafer S. (2015). Comparison of riboflavin and toluidine blue O as photosensitizers for photoactivated disinfection on endodontic and periodontal pathogens in vitro. PLoS ONE.

[34] Fiegler-Rudol J., Zięba N., Turski R., Misiołek M., Wiench R. (2025). Hypericin-Mediated Photodynamic Therapy for Head and Neck Cancers: A Systematic Review. Biomedicines.

[35] Etemadi H., Parker S., Chiniforush N. (2021). Blue light photodynamic therapy with curcumin and riboflavin in the management of periodontitis: A systematic review. J. Lasers Med. Sci..

[36] Alqerban A. (2021). Effectiveness of Riboflavin and Rose Bengal Photosensitizer Modified Adhesive Resin for Orthodontic Bonding. Pharmaceuticals.

[37] Kamran M.A., Qasim M., Udeabor S.E., Hameed M.S., Mannakandath M.L., Alshahrani I. (2021). Impact of riboflavin mediated photodynamic disinfection around fixed orthodontic system infected with oral bacteria. Photodiagnosis Photodyn. Ther..

[38] Moradi M., Fazlyab M., Pourhajibagher M., Chiniforush N. (2022). Antimicrobial action of photodynamic therapy on Enterococcus faecalis biofilm using curing light, curcumin and riboflavin. Aust. Endod. J..

[39] Najari E., Zamani S., Arabi M.S., Ardebili A. (2024). Antimicrobial photodynamic effect of the photosensitizer riboflavin, alone and in combination with colistin, against pandrug-resistant *Pseudomonas aeruginosa* clinical isolates. J. Infect. Chemother..

[40] Alshehri A.H. (2021). Mechanical and antimicrobial effects of riboflavin-mediated photosensitization of in vitro Candida albicans formed on polymethyl methacrylate resin. Photodiagnosis Photodyn. Ther..

[41] Arboleda A., Miller D., Cabot F., Taneja M., Aguilar M.C., Alawa K., Amescua G., Yoo S.H., Parel J.-M. (2014). Assessment of rose bengal versus riboflavin photodynamic therapy for inhibition of fungal keratitis isolates. Am. J. Ophthalmol..

[42] Kashiwabuchi R.T., Carvalho F.R., Khan Y.A., Hirai F., Campos M.S., McDonnell P.J. (2013). Assessment of fungal viability after long-wave ultraviolet light irradiation combined with riboflavin administration. Graefes Arch. Clin. Exp. Ophthalmol..

[43] Sholapurkar A., Sharma D., Glass B., Miller C., Nimmo A., Jennings E. (2020). Professionally Delivered Local Antimicrobials in the Treatment of Patients with Periodontitis—A Narrative Review. Dent. J..

[44] Büchtner A., Meyer U., Kruse-Lösler B., Joos U., Kleinheinz J. (2004). Sustained release of doxycycline for the treatment of peri-implantitis: Randomised controlled trial. Br. J. Oral Maxillofac. Surg..

[45] Micu I.C., Muntean A., Roman A., Stratul Ș.I., Pall E., Ciurea A., Soancă A., Negucioiu M., Barbu Tudoran L., Delean A.G. (2023). A Local Desiccant Antimicrobial Agent as an Alternative to Adjunctive Antibiotics in the Treatment of Periodontitis: A Narrative Review. Antibiotics.

[46] Tomasi C., Koutouzis T., Wennström J. (2008). Locally Delivered Doxycycline as an Adjunct to Mechanical Debridement at Retreatment of Periodontal Pockets. J. Periodontol..

[47] Toledano M., Osorio M.T., Vallecillo-Rivas M., Toledano-Osorio M., Rodríguez-Archilla A., Toledano R., Osorio R. (2021). Efficacy of local antibiotic therapy in the treatment of peri-implantitis: A systematic review and meta-analysis. J. Dent..

